# Letter from Dr. W. A. Hammond

**Published:** 1893-11

**Authors:** William A. Hammond

**Affiliations:** Washington, D. C.


### Correspondence.

### LETTER FROM DR. HAMMOND.

WASHINGTON, D. C. October 21, 1893.

*Editor Texas Medical Journal:*

I am quite sure that you would not do intentional injustice to any one, and yet when you say as you do, in the last number of the TEXAS MEDICAL JOURNAL, that I claim proprietorship in the "Animal Extract," you do me great injustice. I have never claimed anything of the kind. I have given the formulas for their manufacture to the profession and the public, and have repeatedly, in answer to inquiries, explained the details more fully to persons who wished to make them and did not quite understand certain points. I have simply protested against their being made by other than my formulas and falsely called mine. They are not patented, nor are the names trade-marked.

Now, my dear doctor, if you will kindly read the enclosed circulars you will see exactly what stand I have taken.

I have done my best to keep these extracts in a clean course. They have not been advertised in other than medical journals, and the formulas have been made public, and yet I am abused by persons (let us hope ignorantly) as much as Keely, Amick, and others of their stripe.

It is true, the Columbia Chemical Co. did for a very brief period, claim proprietorship in the extracts, but I made them back out of that as soon as I was aware of the fact, and my own utterances have always been to the effect that they were free to the whole world. Now, I ask that you will print this letter in the TEXAS MEDICAL JOURNAL with such remarks as your sense of right calls for, and thus undo frankly and generously the wrong you have done me. Yours sincerely.

WILLIAM A. HAMMOND.

